# Maximal surgical resection and adjuvant surgical technique to prolong the survival of adult patients with thalamic glioblastoma

**DOI:** 10.1371/journal.pone.0244325

**Published:** 2021-02-04

**Authors:** Jaejoon Lim, YoungJoon Park, Ju Won Ahn, So Jung Hwang, Hyouksang Kwon, Kyoung Su Sung, Kyunggi Cho

**Affiliations:** 1 Department of Neurosurgery, Bundang CHA Medical Center, CHA University College of Medicine, Seongnam, Republic of Korea; 2 Institute Department of Biomedical Science, College of Life Science, CHA University, Seongnam, Republic of Korea; 3 Dermay Research Center, Dongtan, Republic of Korea; 4 Department of Neurosurgery, Dong-A University Hospital, Dong-A University College of Medicine, Busan, Republic of Korea; George Washington University, UNITED STATES

## Abstract

The importance of maximal resection in the treatment of glioblastoma (GBM) has been reported in many studies, but maximal resection of thalamic GBM is rarely attempted due to high rate of morbidity and mortality. The purpose of this study was to investigate the role of surgical resection in adult thalamic glioblastoma (GBM) treatment and to identify the surgical technique of maximal safety resection. In case of suspected thalamic GBM, surgical resection is the treatment of choice in our hospital. Biopsy was considered when there was ventricle wall enhancement or multiple enhancement lesion in a distant location. Navigation magnetic resonance imaging, diffuse tensor tractography imaging, tailed bullets, and intraoperative computed tomography and neurophysiologic monitoring (transcranial motor evoked potential and direct subcortical stimulation) were used in all surgical resection cases. The surgical approach was selected on the basis of the location of the tumor epicenter and the adjacent corticospinal tract. Among the 42 patients, 19 and 23 patients underwent surgical resection and biopsy, respectively, according to treatment strategy criteria. As a result, the surgical resection group exhibited a good response with overall survival (OS) (median: 676 days, *p* < 0.001) and progression-free survival (PFS) (median: 328 days, *p* < 0.001) compared with each biopsy groups (doctor selecting biopsy group, median OS: 240 days and median PFS: 134 days; patient selecting biopsy group, median OS: 212 days and median PFS: 118 days). The surgical resection groups displayed a better prognosis compared to that of the biopsy groups for both the O^6^-methylguanine-DNA methyltransferase unmethylated (log-rank *p* = 0.0035) or methylated groups (log-rank *p* = 0.021). Surgical resection was significantly associated with better prognosis (hazard ratio: 0.214, *p* = 0.006). In case of thalamic GBM without ventricle wall-enhancing lesion or multiple lesions, maximal surgical resection above 80% showed good clinical outcomes with prolonged the overall survival compared to biopsy. It is helpful to use adjuvant surgical techniques of checking intraoperative changes and select the appropriate surgical approach for reducing the surgical morbidity.

## Introduction

The thalamus is located deep in the brain and adjacent to important neural structures. The importance of maximal resection in the treatment of glioblastoma (GBM) has been reported in many studies [1–3]. However, the resection of the thalamic tumor is associated with a high rate of morbidity and mortality [4–6], thus maximal resection of thalamic GBM is rarely attempted [7, 8], and the role of maximal surgical resection remains unclear. In the process of thalamic GBM treatment, biopsy is often performed to confirm the pathologic diagnosis and molecular characteristics, while surgical resection remains challenging [1, 8, 9]. Moreover, methods for effective surgical resection have been reported, with some reports including a description of the surgical approach [10, 11].

The purpose of this study was to investigate the role of surgical resection in adult thalamic GBM treatment, as well as to determine the method for achieving maximal radical resection while reducing surgical complications.

## Materials and methods

### Inclusion and exclusion criteria of participants

**Fig 1:**
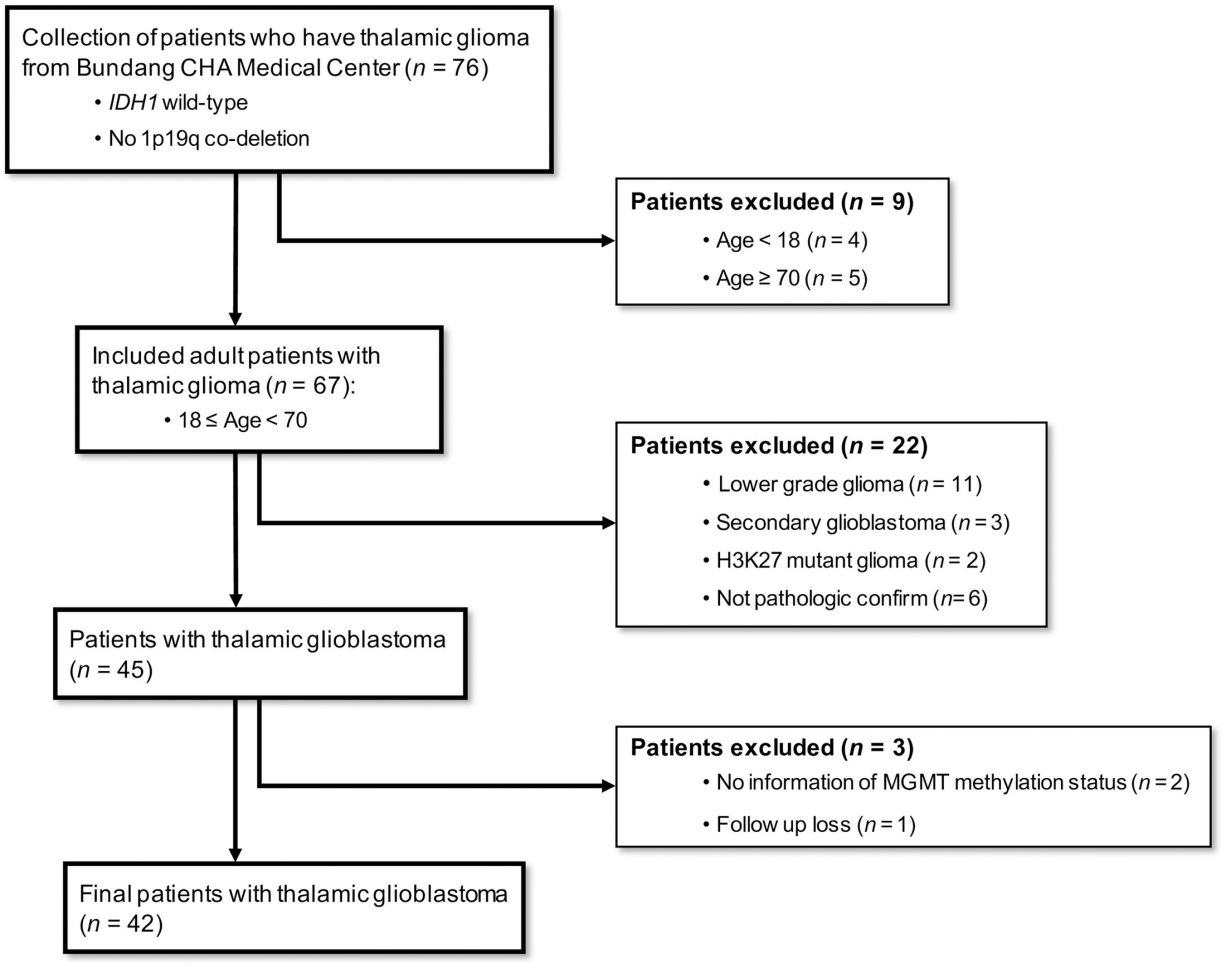
Inclusion and exclusion criteria of participants. From January 2010 to December 2017, the data of 76 patients with thalamic glioma were collected from Bundang CHA Medical Center. We excluded the early on-set patients under 18 years of age (n = 4) and elderly patients above 70 years of age (n = 5). Patients with non-primary glioblastoma, including lower grade gliomas (grade I, II, and III) (n = 11), secondary or recurrent glioblastoma (n = 3), H3K27 mutant glioma (n = 2), and pathologically unclassified tumors (n = 6) were excluded. In addition, we excluded patients for whom there was no information concerning O^6^- methylguanine- DNA methyltransferase methylation status and who were lost to follow-up. Finally, 42 total patients with thalamic glioblastoma were included in this study.

From January 2010 to December 2017, the data of 76 patients with thalamic glioma were collected from Bundang CHA Medical Center. We excluded the early on-set patients under 18 years of age (n = 4) and elderly patients above 70 years of age (n = 5). Because the early on-set patient and elderly patient cohort groups were heterogeneous (e.g., they have different molecular, prognostic, and treatment characteristics), we only included adults in this study. Patients with non-primary GBM including lower grade gliomas (grade I, II and III) (n = 11), secondary or recurrent GBM (n = 3), H3K27 mutant glioma (n = 2) and pathologically unclassified tumors (n = 6) were also excluded. In addition, we excluded patients who did not have information regarding O^6^-methylguanine-DNA methyltransferase (MGMT) methylation status or those who were lost to follow-up. Finally, 42 patients with thalamic GBM were included in this study (Fig 1). This study was approved by the Institutional Review Board of Bundang CHA Medical Center.

### Measurement of clinical information and relevance

Pre- and post-operative magnetic resonance imaging (MRI) scans were reviewed by two neuro-radiologists. Volumetric measurements from immediate postoperative MRI (≤ 48 hour) were used to evaluate the extent of resection. Postoperative tumor status was defined as gross total resection (GTR) if the postoperative T1 contrast-enhanced MRI scans revealed no evidence of residual lesion. The extent of resection was defined as partial resection (PR) (< 80%), subtotal resection (STR) (≥80%, <100%), or total resection based on volumetric analysis of postoperative MRI. Additional variables obtained for analysis included clinical symptoms, molecular characteristics, MGMT methylation status, overall survival (OS), progression-free survival (PFS), and Karnofsky performance status (KPS). The clinical outcomes were analyzed considering these factors.

### Treatment strategies—Patient selection

**Fig 2:**
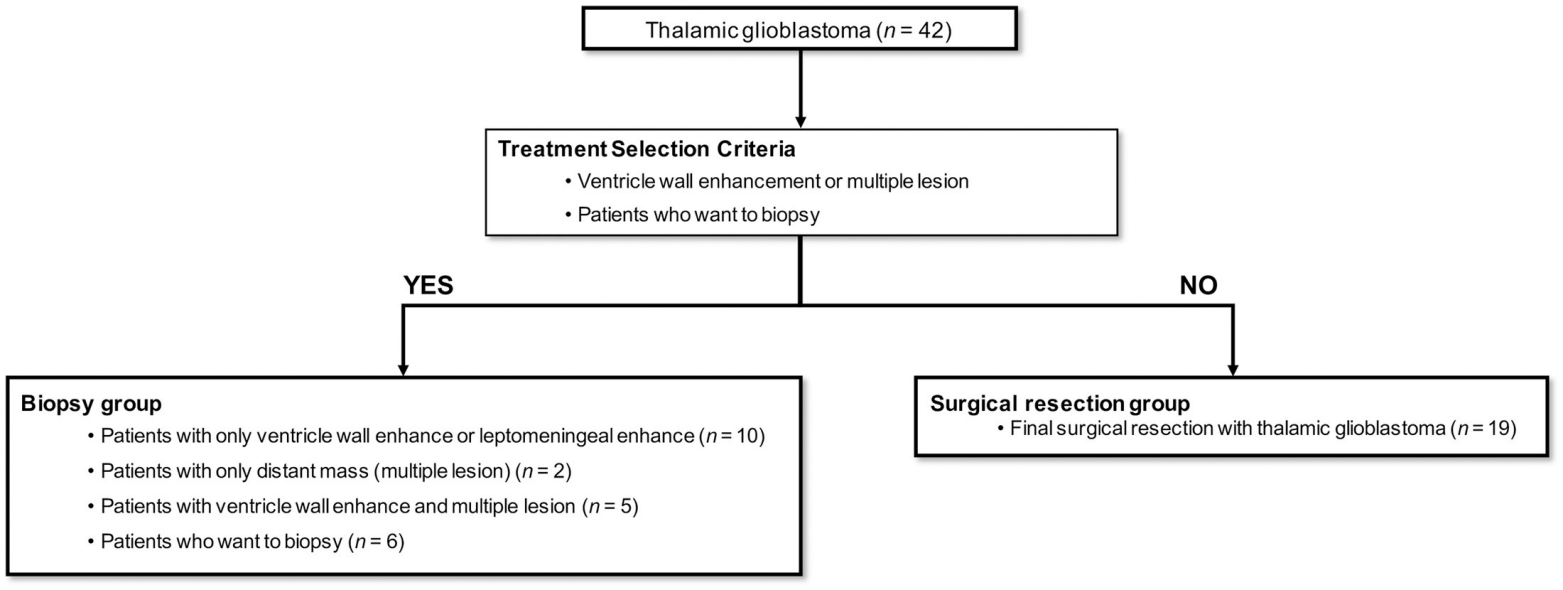
Treatment strategies and patient selection. In cases of suspected thalamic glioblastoma, surgical resection is the treatment of choice in our hospital (n = 19). Biopsy was considered when there was ventricle wall enhancement, leptomeningeal enhancement, or multiple enhancement lesion in a distant location on magnetic resonance imaging (n = 18). Biopsy was performed in cases when the patient or family elected it (n = 6).

In cases where there is suspicion of thalamic GBM, surgical resection was the treatment of choice in our hospital. The aim of surgery is GTR of the tumor if possible. STR was performed in cases for which serious neurological complications were expected, corticospinal tract (CST) damage was suspected, or brainstem injury or vascular injury may have occurred. Biopsy was the treatment choice in patients with ventricle wall enhancement, leptomeningeal enhancement, or multiple enhancement lesion in a distant location (Fig 2). After the pathologic diagnosis was confirmed, all patients in this study were treated with the standard treatment for GBM (concurrent chemo-radiation therapy and temozolomide chemotherapy) [12].

### Adjuvant surgical technique of thalamic GBM

**Fig 3:**
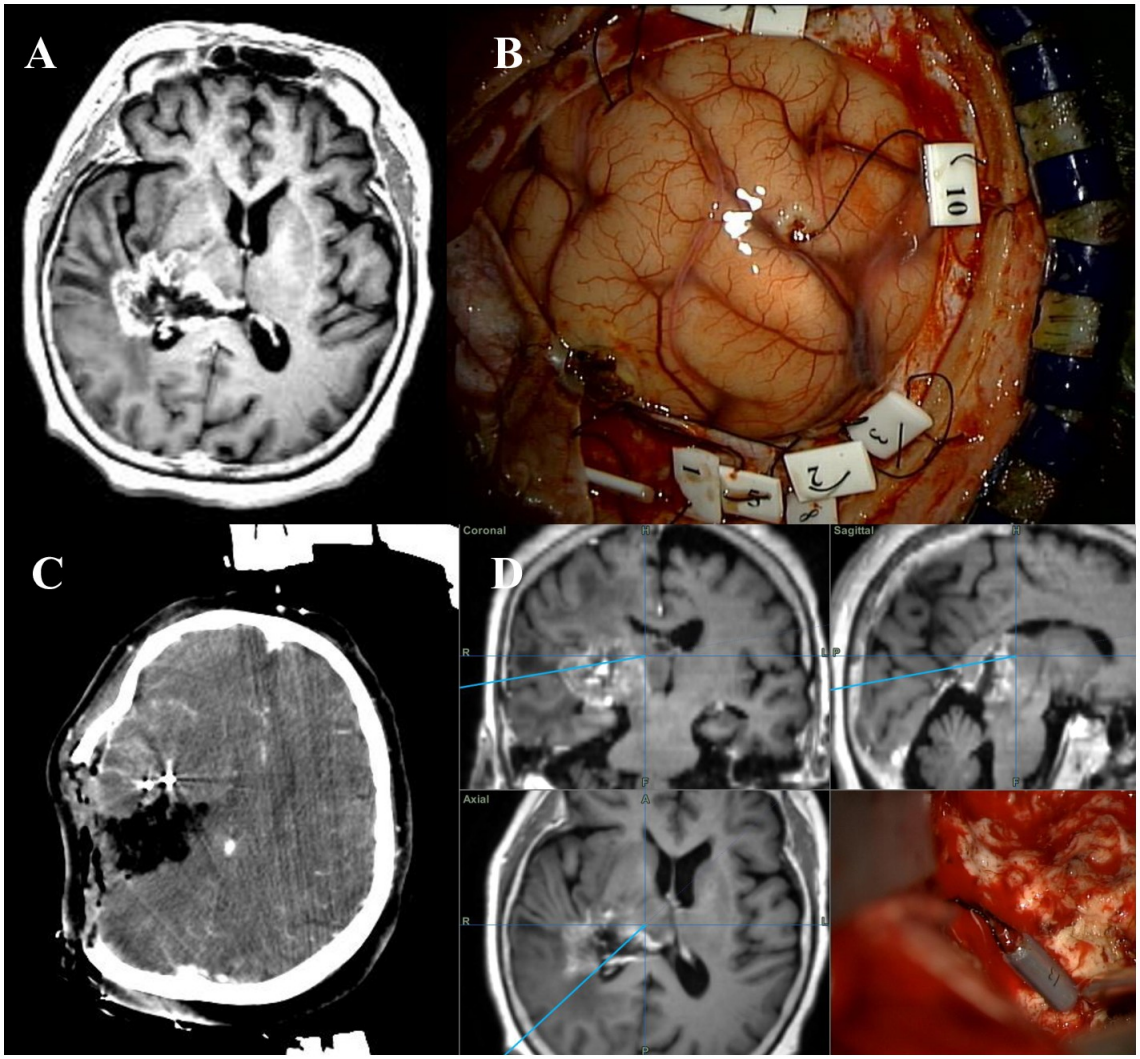
Adjuvant surgical technique. Preoperative magnetic resonance image of a glioblastoma of the right posterior thalamus with lateral extension (A). The tailed bullets are inserted into the target areas (B). During the operation, intraoperative computed tomography image and the tailed bullet technique are used for enabling adjustment for brain shifting and the confirmation of target lesion (C, D).

Preoperative navigation MRI, diffuse tensor tractography imaging (DTI), enhanced computed tomography (CT) were routinely performed and fused together. Tailed bullets were inserted into the target of the tumor through an incision in the small dura incision (<5 mm) before the dura was fully opened (Fig 3B) [13]. The main target regions of the bullets were corticospinal tract and midbrain, which were difficult to discriminate during surgery. The bullet inserted target points were marked in fusion images. During the operation, using these bullets we were able to confirm the target lesion under microscope and compare the fusion image with target point. Because of the reduction in tumor volume during the surgery, the tumor and its surrounding environment were altered; therefore, intraoperative CT was performed to identify shifts in the brain lesion relative to the fusion image [14, 15]. Fluorescence dye (5-ALA: 5-aminolevulinic acid) was used to discriminate the tumorous lesion in all surgical resection cases. Transcranial motor evoked potential (MEP) and monopolar direct subcortical stimulation (DSS) were used to confirm the intraoperative functional status and the location of the CST [16]. Transcranial MEP was monitored at 60~100 mA every 5 minutes throughout the cortical procedure. DSS was initiated at 10 mA and decreased to 6 mA as stimulator approached the CST. If the status of the patient allowed, awake surgery was performed while patients were awake to check the intraoperative functional status of patients.

### Surgical approach

Determination of the surgical approach was selected based on the location of the tumor epicenter and the adjacent corticospinal tract [10, 11]. The shortest corridor from the cortex to the tumors was selected considering the pattern of tumor extension and the location of the CST [10]. A transcortical approach was chosen when the tumor was located at the antero- or posterolateral thalamus and extend in the superior lateral direction. A transcortical-transventricular approach was chosen when the tumor was located at the posterolateral thalamus. When the tumor was located at the medial and posterior superior thalamus, an interhemispheric transcallosal approach was chosen [17, 18], while a transsylvian-transinsular approach was chosen for the lateral thalamic lesions [7]. For the posterior inferior and medial posterior inferior thalamic lesion, an occipital transtentorial approach was adopted. The modified lateral supraorbital (MLSO) approach was used for anterior thalamic tumors [19].

### Statistical analysis

We used t-test, chi-squared test, or Fisher’s exact tests to compare clinical characteristics between the surgical resection group and biopsy group as appropriate. Clinical outcomes, including OS and PFS, were estimated using Kaplan-Meier estimates with a log-rank test and Cox-regression analysis. The statistical power in multivariate Cox-regression analysis was calculated as described in the previous study [20]. We performed a paired Wilcoxon test to confirm the significance of the change in KPS between the surgical resection and biopsy groups. All statistical analyses were performed using RStudio (Version: 1.1.456).

## Results

### Patient characteristics

According to inclusion and exclusion criteria (Fig 1), 42 total patients with primary thalamic GBM were included (S1 Table), 19 of whom underwent surgical resection and 23 of whom received a biopsy according to the selection criteria for patient treatment strategies (Fig 2).

To evaluate factor bias for clinical outcomes (with the exception of surgical resection), differences in several important clinical or molecular factors were analyzed (Table 1). As a result, except for treatment strategies including ventricle wall enhancement and multiple lesions, only Ki-67 was significantly different (*p* = 0.007) between the surgical resection and biopsy groups (Table 1). However, because biopsy was selected in patients with ventricle wall enhancement or multiple lesions, the biopsy group included patients in a poorer health state than patients in the surgical resection group. Therefore, to reduce the selection bias, we stratified biopsy patients into two groups: doctor selecting biopsy group and patient selecting biopsy group (the doctor recommended the surgical resection, but patient selected the biopsy).

**Table 1:** Factors exhibiting clinical relevance.

	Surgical treatment	Biopsy
Age (mean)		
	42.21	50.61
		*p* = 0.053
Tumor volume (mean)		
	26.28	32.13
		*p* = 0.255
Ki-67 proportions (mean)		
	18.95	28.35
		***p* = 0.007**
Tumor components (n)		
Cystic	0	1
Solid	8	12
Solid & cystic	11	10
		*p* = 0.635
Ventricle wall enhancement (n)		
Yes	0	16
No	19	7
		***p* = 2.01E-06**
Multiple lesions (n)		
Yes	0	7
No	19	16
		***p* = 0.011**
Brainstem extension (n)		
Yes	12	8
No	7	15
		*p* = 0.128
Tumor location (n)		
Anterior	3	2
Lateral	3	1
Lateral posterior inferior	6	4
Medial	3	4
Medial posterior inferior	2	5
Posterosuperior	2	7
		*p* = 0.4
Motor symptoms (n)		
Yes	16	23
No	3	0
		*p* = 0.084
Sensory symptoms (n)		
Yes	6	8
No	13	15
		*p* = 1.0
Language symptoms (n)		
Yes	1	3
No	18	20
		*p* = 0.614
Visual symptoms (n)		
Yes	5	5
No	14	18
		*p* = 1.0
Cognitive symptoms (n)		
Yes	10	16
No	9	7
		*p* = 0.421
MGMT status (n)		
Methylated	10	7
Un-methylated	9	16
		*p* = 0.253

Mean values were analyzed using t-test and all other measures were analyzed using the chi-squared or Fisher’s exact tests. **Bold** indicates significant results(p-value<0.05). MGMT, O^6^-methylguanine- DNA methyltransferase

### Clinical outcomes

**Fig 4:**
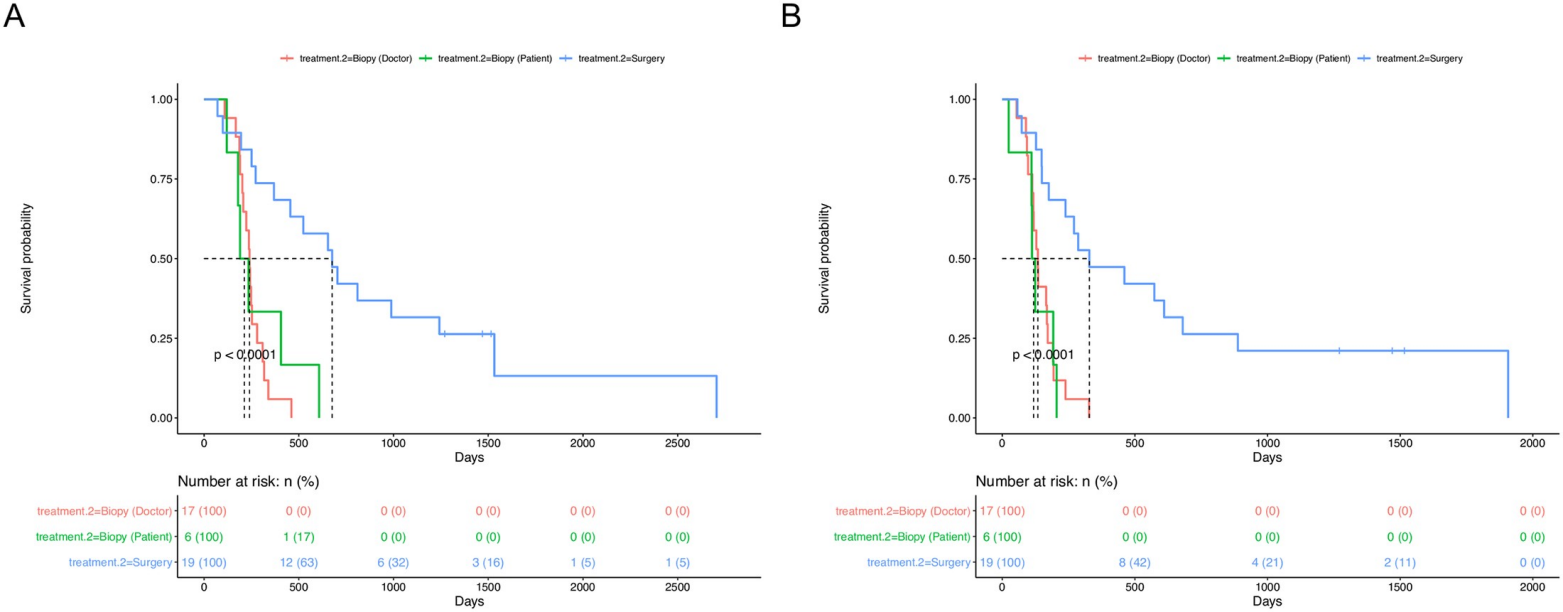
Survival analysis of surgical resection and biopsy groups. A. The mean overall survival time (OS) was significantly longer in patients who underwent surgical resection compared to those who underwent biopsy based on treatment criteria (doctor selecting biopsy group) or those who elected biopsy (patient selecting biopsy group) (*p* < 0.001). B. Progression-free survival in the surgical resection group was significantly longer compared to the doctor selecting biopsy group or the patient selecting biopsy group (*p* < 0.001).

To evaluate the clinical efficacy of surgical resection compared to that of biopsy, we estimated the differences in OS and PFS rates amongst the three groups: surgical resection (n = 19), doctor selected biopsy (n = 17), and patient selected biopsy (n = 6). The surgical resection group exhibited good OS (median: 676 days, *p* = 0.001) (Fig 4A) and PFS (median: 328 days, *p* = 0.001) (Fig 4B) compared with the biopsy groups (doctor selecting biopsy, median OS: 240 days and median PFS: 134 days; patients selecting biopsy, median OS: 212 days and median PFS: 118 days) (Fig 4).

MGMT methylation status is an important factor concerning the prognosis of temozolomide-treated GBM patients [21]. Therefore, we performed survival analysis in each MGMT unmethylated and methylated groups. The surgical resection groups displayed a better prognosis compared to that of the biopsy groups when separated by MGMT methylation status (unmethylated; log-rank *p* = 0.0035 and power: 0.83 (S1A Fig), methylated; log-rank *p* = 0.021 and power: 0.59 (S1B Fig)). The surgical resection group displayed a better prognosis than the unmethylated patient-selecting biopsy group (OS and PFS, log-rank *p* = 0.018 and 0.0054 [S2A and S2B Fig]), but not in the methylated patient-selecting biopsy group (OS and PFS, log-rank *p* = 0.086 and 0.16 [S2C and S2D Fig]).

To evaluate effects of ventricle wall enhancement or multiple lesions on the prognosis of primary thalamic GBM, we compared the difference of the survival rate in biopsy patients with or without ventricle wall enhancement and multiple lesions. As a result, neither multiple lesions (S3A Fig) (log-rank *p* = 0.056) nor ventricle wall enhancement (S3B Fig) (log-rank *p* = 0.33) influenced the prognosis of primary thalamic GBM (S3 Fig).

We also estimated the effect of extent of surgical resections on prognosis of primary thalamic GBM. As a result, within patients in the surgical resection group, there were no significant differences in either PFS or OS (S4 Fig). However, patients who received a GTR tended to have a better PFS than those who received STR (S4A Fig).

Finally, we performed multiple Cox-regression analysis to confirm the effect of surgical resection on the prognosis after adjusting for age, Ki-67 status, preoperative KPS. As a result, only surgical resection was significantly associated with better prognosis (hazard ratio: 0.214, *p* = 0.006, and statistical power: 0.9) (Table 2). The statistical power of multivariate Cox regression survival analysis for surgical resection and biopsy groups was 0.9. Despite the small sample size, a high statistical power was obtained due to the large effect size. The surgical resection group at the age of 30 or older showed better OS (*p* = 0.015) and PFS (*p* = 0.0088) compared to the surgical resection group under 30 years old (S5 Fig). In the surgical resection group, worsening of symptoms after surgery occurred for motor symptoms (10.53%), cognitive symptoms (15.79%), and sensory symptoms (15.79%) (S6 Fig). There were no significant differences in OS in the symptom worsening group (S7 Fig). In motor symptom worsening group, there were significant differences in PFS (*p* = 0.037) (S8 Fig).

**Table 2:** Multiple Cox-regression analysis.

Factor	Hazard ratio	95% CI	p-value
Surgical resection	0.214	0.071–0.646	**0.006**
Age	0.989	0.962–1.017	0.446
Ki-67	1.006	0.976–1.037	0.707
Preoperative KPS	0.979	0.935–1.024	0.356

**Bold** indicates significant result (p-value<0.05), CI; confidence interval, KPS; Karnofsky performance status

Postoperative hemorrhage occurred in 5 surgical resection cases and 3 biopsy cases (*p* = 0.43) (Table 3). There was no need for additional operation. Wound infection occurred in 2 surgical resection cases and resolved with antibiotics medication (*p* = 0.2) (Table 3). Hydrocephalus occurred in 3 surgical resection cases and treated with ventriculoperitoneal shunt (*p* = 0.08) (Table 3). Operation-related neurologic complications were provided for detailed clinical characteristics and durations (Table 3). In the group that underwent surgery, there were 7/19 cases in the improved KPS group, 5/19 cases in the no change in KPS group, and 7/19 cases in the worse KPS group. In the worse KPS group, there were 2/7 cases in which the KPS was worsened by 20 or more compared to the preoperative status, and in 1 case, severe daily living restrictions were caused with KPS less than 60. In the entire biopsy group, there were 0/23 cases in the improved KPS group, 19 cases in the no change in KPS group, and 4 cases in the worse KPS group. The KPS worsened due to biopsy by 10 points in all 4 cases (Table 3).

**Table 3:** Distribution of surgical and neurological complications between surgical resection and biopsy group.

	Surgical resection (n = 19)	Biopsy (n = 23)	p-value*
**Surgical complication**			
Post-operative hemorrhage	5	3	0.43
Re-operation d/t hemorrhage	0	0	1
Infection	2	0	0.2
Hydrocephalus	3	0	0.08
**Neurological complication related to surgery**			
*Motor weakness*	6	5	0.5
Transient	2	4	
Permanent	4	1	p* = 0.24
*Sensory deficit*	6	4	0.47
Transient	3	3	
Permanent	3	1	p* = 0.57
*Visual deficit*	3	5	0.71
Transient	2	5	
Permanent	1	0	p* = 0.38
*Cognition worsening*	4	1	0.16
Transient	3	1	
Permanent	1	0	p* = 1
*KPS worsening*	7	4	0.18
Transient	4	4	
Permanent	3	0	p* = 0.24

p-value*; Calculated using Fisher’s exact test, p*; Fisher’s exact test for symptom duration within each neurological complication between surgical resection and biopsy groups

There was no significant difference in surgery-related neurological complications such as motor weakness, sensory deficit, visual deficit, cognition worsening, and KPS worsening between the surgical resection and the biopsy groups (*p* > 0.5) (Table 3).

### Adjuvant surgical technique and surgical approach

In all surgical resection cases, navigation MRI, DTI, enhanced CT, intraoperative CT, tailed bullet, transcranial MEP, and DSS were used, as well as fluorescence dye (5-aminolevulinic acid) (Fig 3, S9 Fig). Awake surgery was performed in five cases when patient status and surgical position allowed. A total of six surgical approaches were applied (S10 Fig). The transcortical transventricular approach was the most commonly used (6/19) and applied to lateral posterior inferior and posterior superior thalamic lesions. The transcortical approach was used in five cases and applied to anterior, lateral, and lateral posterior inferior thalamic lesions. The interhemispheric transcallosal approach was used in four cases and applied to medial and posterior superior lesions. The occipital transtentorial approach was used in two cases and applied to medial posterior inferior lesions. The transsylvian-transinsular approach was used in one case for a lateral thalamic lesion. The MLSO approach was used in one case for an anterior thalamic lesion. There was no significant difference in OS (*p* = 0.69) and PFS (*p* = 0.55) according to surgical approach.

### Concise case report

A 29-year-old woman was admitted to our hospital with confusion mentality and motor weakness. Preoperative MRI showed a posterior inferior thalamic contrast-enhancing mass with lateral extension (Fig 3A). After craniotomy was performed, the bullet was inserted into several target lesions (Fig 3B). During operation, fluorescence dye (5-ALA) was used to identify the tumor lesion (S9C and S9D Fig). The preoperative MRI image was fused with an intraoperative CT image (Fig 3C, S9E and S9F Fig) to adjust for brain shifting and to confirm the target lesion (Fig 3D, S9E and S9F Fig). The post-operative MRI showed the GTR of the tumor (S3B Fig).

## Discussion

GBM is a malignant tumor for which patient have a short survival time despite surgical treatment and chemo-radiation therapy. The importance of surgical treatment in GBM patients has been reported in many studies [1–3, 22]. In particular, there was a significant difference in survival time according to the degree of resection, and the importance of maximal resection was emphasized [1, 2, 9]. Recently, studies have shown that surgical resection, including not only the contrast-enhancing lesion but also the signal-changed lesion in FLAIR images, increases the survival time [23, 24]. In addition, the concept of supra-total resection, which removes not only the region showing signal change on the MRI but also the region associated with tumors, has been reported and showed a survival benefit [25–28].

For thalamic GBM, even the choice of surgical resection is difficult [5]. In large series of thalamic GBM, surgical resection was performed in 10 cases among 57, most of which were treated with biopsy followed by chemotherapy and radiation therapy [8]. In our hospital, surgical resection has been the treatment of choice when thalamic GBM is suspected. Biopsy is selected when there is suspicion of cerebrospinal fluid space spreading due to ventricle wall-enhancing lesion or leptomeningeal-enhancing lesion or multiple tumorous conditions where the mass appears in distant lesions (Fig 2). When comparing the survival outcome, the surgical resection group had significantly longer OS and PFS than the biopsy group (*p* < 0.001) (Fig 4). Because biopsy was used for patients with either ventricle wall enhancement or multiple lesions, the biopsy group had a poorer prognosis than the non-biopsy group; therefore, this group may have had selection bias for high Ki-67 and poor prognosis (Table 1). Thus, to solve the problem of selection bias, we performed subgroup analysis that stratified patients into a doctor selecting biopsy group and a patient selecting group and compared these groups to the surgical resection group. Both OS and PFS were significantly longer in the surgical resection group (*p* < 0.001) (Fig 4). There were significant differences in survival when comparing the surgical resection group and the patient-selecting biopsy group (*p* < 0.001) (Fig 4). So, if surgical resection was performed in this patient selection biopsy group, it could be more helpful for patient survival. This analysis implies that surgical resection may have been helpful to the thalamic GBM patients with no ventricular seeding or no multiple lesion. In MGMT unmethylated thalamic GBM patients, findings revealed that surgical resection could be more helpful than biopsy (OS and PFS, log-rank *p* = 0.018 and 0.0054 [S2A and S2B Fig]). In MGMT methylated thalamic GBM patients, OS and PFS between surgical resection and patient selected biopsy groups were not significantly different (OS and PFS, log-rank *p* = 0.086 and 0.16, respectively) (S2C and S2D Fig).

Maximal resection of the contrast-enhancing lesion in thalamic GBM is difficult. In the study of Esquenazi et al., all cases of surgery were subtotal resection [8]. In a study by Kiran et al., GTR or near total resection was achieved in seven cases among 12 patients with thalamic GBM, which is a relatively high rate [10]. However, the proportion of GTR is not high in most papers that have published surgical results concerning thalamic GBM [7, 8, 18]. In the present study, GTR was achieved in 11 among 19 patients who underwent surgical resection (S1 Table). However, there were no significant differences in OS (*p* = 0.33) and PFS (*p* = 0.38) between the GTR and STR groups (S5 Fig). When evaluating the postoperative results, STR was defined as 80% to 100% resection; therefore, when compared the results of STR to biopsy, surgical resection group had a significantly longer OS and PFS than did the biopsy groups (Fig 4). Therefore, maximal resection of more than 80% is thought to have a better survival benefit than non-maximal resection. Even in the STR group, it was better to attempt aggressive surgical treatment, as it showed a significant difference in OS (*p* = 0.0017) and PFS (*p* = 0.0084) when compared to the biopsy group (S5C and S5D Fig).

**Fig 5:**
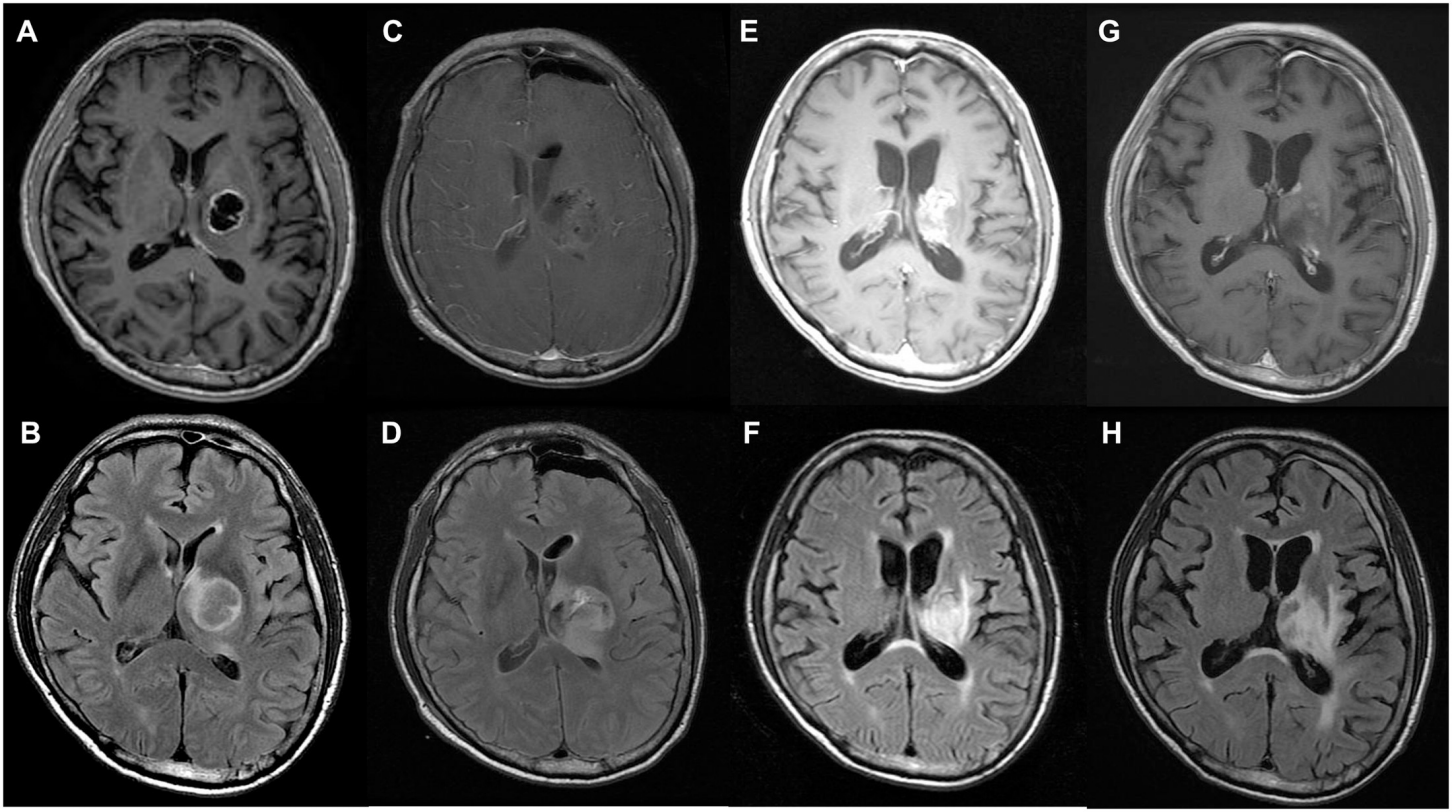
Illustrative case 1. A 68-year-old man was admitted to our hospital with headache and blurred vision. Preoperative magnetic resonance imaging (MRI) showed a cystic and solid mass in a lateral thalamic lesion on T1-contrast enhanced (A) and T2-fluid-attenuated inversion recovery (FLAIR) high signal imaging (B). Postoperative MRI showed gross total resection of the tumor in T1-contrast enhanced images (C) and subtotal resection of the tumor in T2-FLAIR images (D). At the one-year follow-up MRI, there was irregular and fuzzy enhancement in T1-contrast-enhanced images (E) and a high signal in T2-FLAIR images (F). At the 2-year follow-up MRI, there was a stable state in T1-contrast enhanced images (G) and T2-FLAIR images (H). The patient is still alive, and the postoperative survival time is 1515 days.

**Fig 6:**
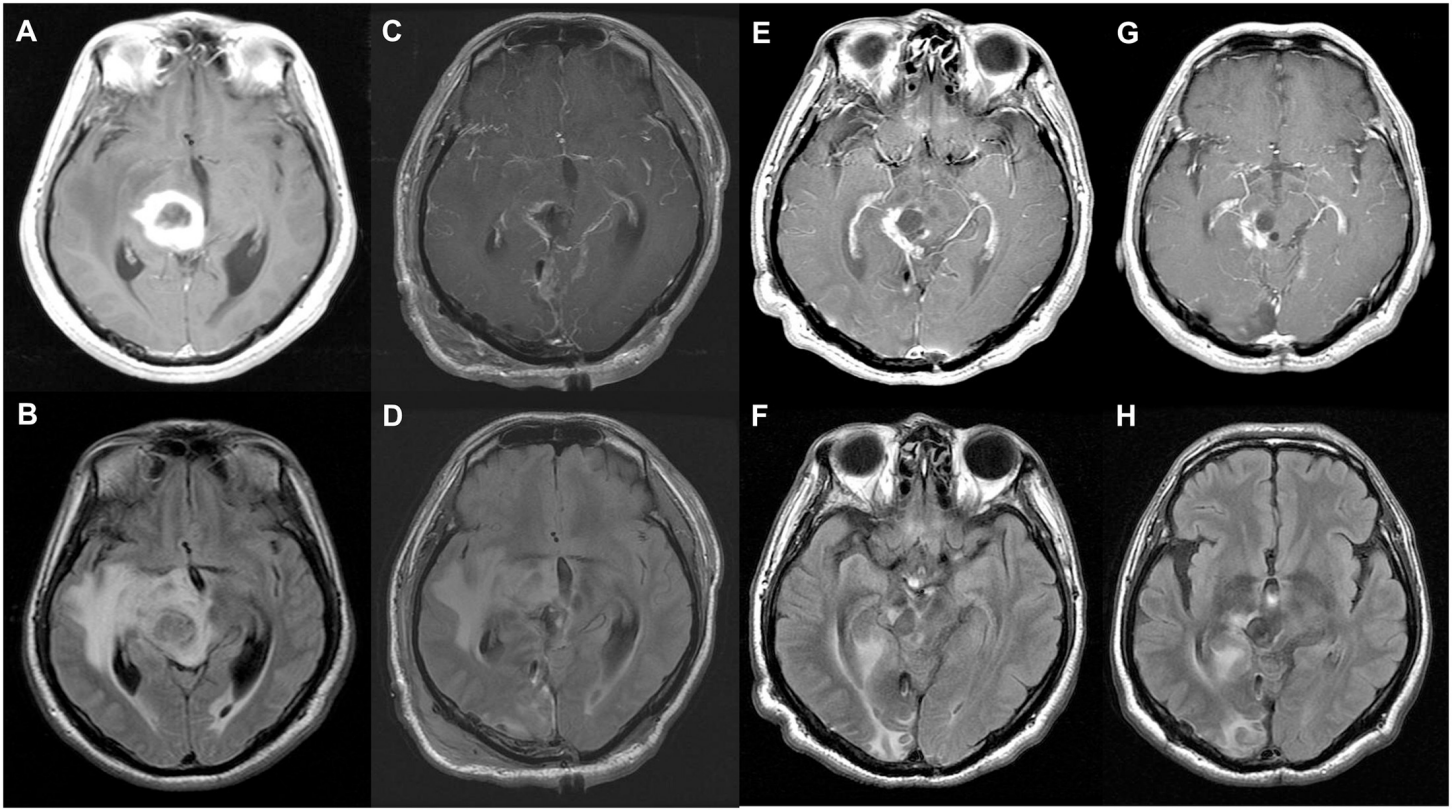
Illustrative case 2. A 52-year-old woman was admitted to our hospital with confused mentality, diplopia and motor weakness. Preoperative magnetic resonance imaging (MRI) showed a cystic and solid mass in a medical posterior inferior thalamic lesion on T1-contrast enhanced (A) and T2-FLAIR high signal imaging (B). Postoperative MRI showed gross total resection of the tumor on T1-contrast enhanced images (C) and subtotal resection of the tumor on T2-FLAIR images (D). At the one-year follow up MRI, there was irregular enhancement on T1- contrast enhanced images (E) and high signal on T2-FLAIR images (F). At the 2-year follow-up MRI, there was a stable state on T1-contrast enhanced (G) and T2- FLAIR images (H). The patient is still alive, and the postoperative survival time is 1469 days.

Based on FLAIR images, there was no case of GTR in this study (S1 Table). However, it was difficult to remove all of the high signal lesions in FLAIR images, as these lesions spread over the thalamus or around the brainstem and CST (Figs 5 and 6). Although not all FLAIR signal-changed lesions can be removed, maximal resection of contrast-enhancing lesions can increase the survival of patients with thalamic GBM.

In addition to maximal resection of the tumor, functional preservation is also important. The thalamus is associated with various body functions, and thalamus GBM surgical resection can cause severe neurologic deficits [4, 8]. Thalamic GBM surgical complications are also related to poor prognosis. It is difficult to distinguish margins of the thalamic GBM from normal tissue during surgery, and it is more difficult to judge the tumor boundary when structure displacement occurs during surgery. The current study indicated that adjuvant surgical techniques, including the use of a tailed bullet and intraoperative CT, can help delineate the target lesion [13–15]. When the bullet was encountered where the boundary was unclear during surgery, the bullet could show not only the target lesion, but also the degree of movement of the structure (Fig 3). In addition, intraoperative CT during surgery can be used to confirm the change of the existing preoperative MRI image and correct the position by checking the bullet position. While intraoperative MRI is more useful during thalamic GBM surgery [29, 30], our institution only employs intraoperative CT, thus it was difficult to confirm the T2 signal-changed lesion or the FLAIR high- signal lesion. After the tailed bullet was inserted into the target lesion (T2 or FLAIR signal changed lesion), it helped to adjust for intraoperative displacement of the lesion by allowing comparison of the preoperative and intraoperative images. Moreover, fluorescence dye was used to discriminate the tumorous lesion in all surgical resection cases [31, 32], and awake surgery, which is helpful for prevention of neurological complications, [33, 34] was performed in five cases. The usefulness of fluorescence dye (5-ALA) has already been presented in numerous papers, and many institutions use 5-ALA for glioblastoma surgery. We also use 5-ALA as an aid to help differentiate tumorous lesions. Since only 5 cases of awake surgery was performed, it was difficult to analyze the surgical impact of awake surgery in this study. The surgical technique described above was used in all thalamic GBM surgical resection, and the clinical outcomes and postoperative complication rate was good compared to surgical results in other papers [5, 7, 8, 10, 35–39] (S6 Fig, Table 3). There were no significant differences in surgery-related complications, including postoperative hemorrhage, infection, hydrocephalus and neurological complications, between the surgical resection group and the biopsy group (Table 3). There were no significant differences in OS in the symptoms worsening group (S7 Fig), but significant differences in PFS (S8 Fig). Therefore, the adjuvant technique used during surgery in the present study helped to achieve good results and longer survival in thalamic GBM surgery.

Since thalamic GBM surgery is very likely to induce high morbidity, the functional status of the patient after surgery is extremely important. If the survival was increased by surgery but the functional deficit was severe, the role of surgery would have been very limited. Of the 19 patients who underwent surgical resection, 18 were able to maintain or improve their functional status with a KPS score of over 60 without suffering severe neurologic deficit. Among the 19 patients in the surgical resection group, a worsening KPS score of more than 20 and less than 60 points was observed only in 1. As a result, there was no significant difference in worsening of KPS between surgical resection and biopsy groups (*p* = 0.18). We identified that using surgical resection to treat adult thalamic GBM prolonged the survival and maintained the performance status of patients.

In our studies, the most important aspect of approach selection in thalamic tumor surgery is saving normal brain structures. We select the approach that can minimize damage to the CST and other structures by accurately identifying the thalamus epicenter corresponding to the tumor origin and the positional relationship with important connected neural structures. Ranger-Castilla et al. have provided an excellent classification of six recommended surgical approaches for six different regions [11]. Based on this classification, each approach is selected according to the tumor origin location and extension pattern [10]. If possible, it is best to avoid CST in the surgical corridor and to choose an approach that minimizes damage to the language cortex and visual pathways. There was no difference in OS (*p* = 0.69) and PFS (*p* = 0.55) according to surgical approach. In addition, thalamic GBMs are deep-seated tumors; hence different approaches are selected depending on the surgeon’s choice to prevent retraction injury during surgery. For a posterior location, D’Angelo et al. recommend the posterior interhemispheric parasplenial approach [40], while Steiger et al. preferred the contralateral infratentorial supracerebellar approach for the medial aspect of the pulvinar [41]. We prefer an occipital transtentorial approach because of the relatively wide view, as a retraction effect can be expected using natural gravity, thus avoiding damage to the visual field in case of a medial posterior inferior thalamic lesion. The surgical approach was selected based on these strategies, and good results were obtained in this study. The degree of surgical resection and the possibility of neural structure injury may significantly differ for each approach, and thus the approach should be selected after considering the purpose of surgery.

This is an observational study with a retrospective design. The selection bias existed in making comparisons between biopsy and surgical resection group, so we performed the additional subgroup analysis. Since this study contains only a relatively small number of surgical resection cases, further research with a larger number is needed. The H3K27m mutation is an important prognostic factor for thalamic GBM [42, 43], but it has not been included in this study because it has only been tested in a very small number of cases in our institution. More molecular diagnosis should be included in future studies.

## Conclusions

In patients with thalamic GBM, it is important to select appropriate candidates for surgical resection. If there is no ventricle wall-enhancing lesion or there are no multiple lesions, longer survival can be expected than that of biopsy alone when maximal surgical resection is above 80%. It is helpful to use tailed bullet and intraoperative image modality to reduce surgical morbidity, and it is important to check the neurophysiologic state through careful monitoring, such as MEP and DSS. The surgical approach might be selected on the basis of the location of the tumor epicenter and the adjacent CST.

## Supporting information

S1 Table Detailed demographic of patients. (PDF)

S1 Fig KM-plots showing difference between surgical resection and each biopsy condition group with overall survival within the MGMT un-methylated group (a), and the methylated group (b). (DOCX)

S2 Fig KM-plots showing difference between surgical resection and patient selecting biopsy group with overall survival and progression-free survival within the MGMT un-methylated group (a, b), and the methylated group (c, d). (DOCX)

S3 Fig KM-plots showing difference in overall survival with or without multiple lesions (a) and ventricle wall enhancements (b) within the biopsy only group. (DOCX)

S4 Fig KM-plots showing difference in overall survival (a) and progression-free survival (b) between GTR and STR within the surgical resection only group, overall survival (c) and progression-free survival (d) between the STR group and the biopsy group. (DOCX)

S5 Fig KM plots of comparison in terms of overall (a) or progression-free (b) survival between patients who were above and below at 30 years old within the surgical resection group. (DOCX)

S6 Fig Pie charts of motor (a), cognitive (b), visual (c) or sensory symptom (d) change from the preoperative status to the postoperative status. Yes to No; score less than Grade 5 of motor symptom or presence of other symptoms at the preoperative state but improved after operation, Yes to Yes; score less than Grade 5 of motor symptom or presence of other symptoms at the preoperative state and no change in the state after operation, No to Yes; Grade 5 of motor symptom or without other symptoms at the preoperative state but occurred after operation, No to No; Grade 5 of motor symptom or without any another symptom at both the pre- and postoperative states.
(DOCX)

S7 Fig KM plots showing overall survival curves of postoperative sensory (a), motor (b), visual (c), and cognitive (d) symptom worsening. (DOCX)

S8 Fig KM plots showing progression-free survival curves of postoperative sensory (a), motor (b), visual (c), and cognitive (d) symptom worsening. (DOCX)

S9 Fig Preoperative and postoperative magnetic resonance image of a glioblastoma of the right posterior thalamus with lateral extension (A, B). Fluorescence dye (5-aminolevulinic acid) imaging of the posterior thalamus with lateral extension (C, D). Preoperative magnetic resonance image was fused with an intraoperative computed tomography image and use of the tailed bullet technique during the operation for enabling adjustment for brain shifting and the confirmation of target lesion (E, F).
(DOCX)

S10 Fig A diagram for surgical approach according to location of thalamic tumor lesions. (DOCX)
