# Supplementary material for: Maximal surgical resection and adjuvant surgical technique to prolong the survival of adult patients with thalamic glioblastoma
Source: PLoS One. 2021 Feb 4;16(2):e0244325. doi: 10.1371/journal.pone.0244325 (PMC7861362; doi:10.1371/journal.pone.0244325)
Supplement: S1 Table — (PDF) [file pone.0244325.s001.pdf]

Table S1 Clinical information of participants

Patients ID	Age	Sex	Treatment*	Tumor volume	Tumor component	ventricle wall enhance	multiple lesion	brain stem extension	Location	Surgical method	Extent of resection within enhance region by MR image	Extent of resection within T2 Flair by MR image	Preop. language Sx.*	Preop. sensory Sx. (aggrevation after postop.)*	Preop. visual Sx. (aggrevation after postop.)*	Preop. cognitive Sx. (aggrevation after postop.)*	Motor (change to postop.)*	KPS (change to postop.)*	OS time*	OS*	PFS time*	PFS*	MGMT status	Ki 67 (%)
1	46	M	Surgery	22.61	Solid & cystic	No	No	No	Anterior	Transcortical	STR	STR	No	No (No)	No (No)	No (No)	4 (+1)	80 (+10)	704	1	610	1	Un-methylation	17
2	45	M	Surgery	8.24	Solid & cystic	No	No	No	Anterior	MLSO	GTR	STR	No	No (No)	No (No)	No (No)	5 (-1)	90 (-10)	455	1	286	1	Un-methylation	14
3	52	F	Surgery	5.32	Solid & cystic	No	No	Yes	Medial posteior inferior	Occipital transtentorial	GTR	STR	No	Yes (Yes)	Yes (No)	Yes (No)	4 (+1)	70 (+10)	1469	0	1469	0	Methylation	18
4	60	F	Surgery	27.3	Solid	No	No	Yes	Medial	Interhemispheric transcallosal	GTR	STR	No	Yes (No)	No (No)	No (Yes)	4 (-1)	80 (-10)	369	1	238	1	Un-methylation	10
5	20	M	Surgery	23.2	Solid & cystic	No	No	Yes	Medial	Interhemispheric transcallosal	STR	STR	No	No (No)	Yes (Yes)	Yes (Yes)	4 (-1)	70 (-10)	654	1	460	1	Methylation	5
6	27	M	Surgery	15.9	Solid & cystic	No	No	Yes	Medial posteior inferior	Occipital transtentorial	STR	STR	No	No (No)	No (No)	Yes (No)	4 (+0)	70 (+0)	272	1	175	1	Methylation	15
7	30	M	Surgery	17.2	Solid	No	No	No	Lateral	Transsylvian-transinsular	GTR	STR	No	Yes (Yes)	No (No)	Yes (Yes)	3 (+0)	60 (+0)	988	1	888	1	Un-methylation	21
8	68	M	Surgery	6.87	Solid & cystic	No	No	No	Lateral	Transcortical	GTR	STR	No	No (No)	Yes (Yes)	No (No)	5 (+0)	80 (+20)	1515	0	1515	0	Methylation	8
9	41	M	Surgery	19.96	Solid	No	No	Yes	Lateral posteior inferior	Transcortical transventricular	STR	STR	No	No (Yes)	No (No)	No (No)	4 (+1)	80 (+10)	2705	1	1907	1	Un-methylation	11
10	20	M	Surgery	36.52	Solid & cystic	No	No	Yes	Posterosuperior	Transcortical transventricular	GTR	STR	No	Yes (No)	No (No)	Yes (No)	2 (+1)	60 (+10)	99	1	73	1	Methylation	21
11	29	F	Surgery	31.27	Solid & cystic	No	No	Yes	Lateral posteior inferior	Transcortical transventricular	GTR	STR	No	No (No)	No (No)	Yes (No)	4 (+0)	70 (+10)	251	1	148	1	Methylation	35
12	36	M	Surgery	29.94	Solid	No	No	Yes	Lateral posteior inferior	Transcortical transventricular	PR	PR	Yes	No (Yes)	No (No)	Yes (No)	4 (+0)	70 (+0)	195	1	127	1	Un-methylation	18
13	45	M	Surgery	22.4	Solid	No	No	No	Lateral posteior inferior	Transcortical transventricular	GTR	STR	No	No (No)	No (No)	No (Yes)	4 (-1)	80 (-10)	1242	1	328	1	Methylation	19
14	36	M	Surgery	68.21	Solid & cystic	No	No	Yes	Lateral posteior inferior	Transcortical transventricular	GTR	STR	No	Yes (No)	Yes (Yes)	Yes (No)	3 (+0)	70 (+0)	1532	1	680	1	Methylation	40
15	50	F	Surgery	25.1	Solid	No	No	No	Lateral	Transcortical	GTR	STR	No	No (No)	No (No)	No (No)	4 (+0)	80 (+0)	810	1	573	1	Un-methylation	21
16	56	F	Surgery	65.41	Solid & cystic	No	No	Yes	Lateral posteior inferior	Transcortical	STR	STR	No	Yes (Yes)	Yes (Yes)	Yes (Yes)	3 (-2)	70 (-30)	71	1	57	1	Un-methylation	30
17	67	F	Surgery	36.4	Solid	No	No	No	Anterior	Transcortical	GTR	STR	No	No (No)	No (No)	Yes (No)	4 (+0)	70 (+10)	1270	0	1270	0	Methylation	13
18	53	F	Surgery	21.5	Solid	No	No	Yes	Posterosuperior	Interhemispheric transcallosal	STR	STR	No	No (Yes)	No (No)	No (No)	4 (+0)	70 (-10)	524	1	270	1	Un-methylation	29
19	21	F	Surgery	15.9	Solid & cystic	No	No	Yes	Medial	Interhemispheric transcallosal	STR	STR	No	No (No)	No (No)	No (Yes)	5 (-2)	90 (-20)	676	1	149	1	Methylation	15
20	63	M	Biopy (Patient)	62.4	Solid	No	No	No	Lateral posteior inferior	-	-	-	Yes	No (No)	No (No)	Yes (No)	4 (+0)	60 (+0)	179	1	124	1	Un-methylation	32
21	39	M	Biopy (Patient)	57.9	Solid	No	No	Yes	Posterosuperior	-	-	-	No	No (Yes)	No (No)	Yes (No)	3 (+0)	60 (+0)	235	1	192	1	Methylation	28
22	36	M	Biopy (Patient)	11	Solid	No	No	No	Posterosuperior	-	-	-	No	No (No)	No (No)	No (No)	4 (+0)	70 (+0)	190	1	24	1	Un-methylation	28
23	56	F	Biopy (Patient)	21.55	Solid & cystic	No	No	No	Posterosuperior	-	-	-	No	Yes (No)	No (No)	Yes (No)	4 (+0)	60 (+0)	406	1	205	1	Un-methylation	18
24	60	F	Biopy (Patient)	9.97	Cystic	No	No	No	Posterosuperior	-	-	-	No	No (No)	No (No)	No (No)	4 (+0)	70 (+0)	607	1	110	1	Methylation	21
25	65	M	Biopy (Patient)	30.86	Solid	Yes	No	No	Medial posteior inferior	-	-	-	No	No (Yes)	No (No)	Yes (Yes)	4 (+0)	60 (+0)	120	1	112	1	Un-methylation	40
26	38	M	Biopy (Doctor)	52.9	Solid	Yes	No	No	Medial	-	-	-	No	No (No)	No (No)	Yes (No)	3 (+0)	50 (+0)	237	1	134	1	Methylation	25
27	56	M	Biopy (Doctor)	18.9	Solid	Yes	Yes	No	Lateral posteior inferior	-	-	-	No	No (No)	No (No)	No (No)	4 (+0)	70 (+0)	187	1	117	1	Un-methylation	35
28	42	M	Biopy (Doctor)	52.6	Solid & cystic	Yes	No	No	Medial	-	-	-	No	No (No)	No (No)	Yes (No)	3 (+0)	60 (+0)	207	1	168	1	Methylation	18
29	52	F	Biopy (Doctor)	48.7	Solid & cystic	Yes	Yes	Yes	Medial posteior inferior	-	-	-	Yes	Yes (Yes)	Yes (Yes)	Yes (Yes)	3 (-1)	50 (+0)	109	1	89	1	Un-methylation	21
30	40	M	Biopy (Doctor)	37.3	Solid & cystic	Yes	Yes	Yes	Medial	-	-	-	No	Yes (No)	No (No)	Yes (No)	3 (+0)	60 (-10)	202	1	93	1	Un-methylation	70
31	41	F	Biopy (Doctor)	20.6	Solid & cystic	Yes	No	No	Anterior	-	-	-	No	No (No)	No (No)	Yes (No)	4 (+0)	50 (+0)	309	1	118	1	Un-methylation	45
32	65	M	Biopy (Doctor)	39.3	Solid	No	Yes	Yes	Lateral posteior inferior	-	-	-	No	No (Yes)	No (No)	No (Yes)	4 (-1)	60 (+0)	190	1	97	1	Un-methylation	15
33	57	F	Biopy (Doctor)	16.8	Solid	Yes	No	No	Posterosuperior	-	-	-	No	Yes (No)	No (No)	No (No)	4 (+0)	70 (+0)	280	1	54	1	Un-methylation	22
34	42	F	Biopy (Doctor)	31.1	Solid & cystic	Yes	No	Yes	Medial posteior inferior	-	-	-	No	Yes (Yes)	Yes (No)	Yes (Yes)	3 (-1)	50 (+0)	253	1	135	1	Un-methylation	31
35	54	F	Biopy (Doctor)	38.08	Solid & cystic	Yes	No	No	Lateral posteior inferior	-	-	-	No	No (Yes)	Yes (Yes)	Yes (No)	4 (-1)	60 (+0)	339	1	238	1	Methylation	23
36	58	F	Biopy (Doctor)	37.82	Solid	Yes	No	No	Posterosuperior	-	-	-	No	No (No)	No (Yes)	No (No)	4 (+0)	70 (-10)	240	1	193	1	Un-methylation	25
37	55	M	Biopy (Doctor)	43.96	Solid & cystic	Yes	No	Yes	Lateral	-	-	-	Yes	Yes (No)	No (Yes)	Yes (No)	4 (+0)	60 (+0)	248	1	113	1	Un-methylation	35
38	47	M	Biopy (Doctor)	33.81	Solid	Yes	No	Yes	Posterosuperior	-	-	-	No	No (No)	No (Yes)	No (No)	4 (+0)	70 (-10)	461	1	327	1	Methylation	40
39	23	M	Biopy (Doctor)	9.85	Solid	No	Yes	No	Medial	-	-	-	No	Yes (No)	No (Yes)	Yes (Yes)	3 (+0)	60 (+0)	317	1	191	1	Un-methylation	23
40	63	M	Biopy (Doctor)	20.5	Solid	Yes	No	Yes	Medial posteior inferior	-	-	-	No	No (No)	No (Yes)	Yes (No)	3 (+0)	50 (+0)	223	1	171	1	Un-methylation	17
41	58	F	Biopy (Doctor)	20.86	Solid & cystic	Yes	Yes	No	Medial posteior inferior	-	-	-	No	No (No)	Yes (Yes)	Yes (No)	3 (+0)	60 (+0)	243	1	165	1	Methylation	15
42	54	M	Biopy (Doctor)	22.24	Solid & cystic	Yes	Yes	No	Anterior	-	-	-	No	Yes (Yes)	Yes (No)	Yes (Yes)	3 (-1)	60 (-10)	167	1	128	1	Un-methylation	25

Column name	Description	Indicate
**Treatment***	Surgical treatment or biopsy	Biopsy (Patient); Biopsy by patient request, Biopsy (Doctor); Biopsy by doctor's decision
**Preop. language Sx.***	Language symptom at preoperation	-
**Extent of resection within enhance region by MR image**	Coresponding to T1 enhancing region	GTR; Gross total resection, STR; Sub-total resection, PR; Partial resection
**Extent of resection within T2 Flair by MR image**	Coresponding to T2 Flair enhancing region	GTR; Gross total resection, STR; Sub-total resection, PR; Partial resection
**Preop. sensory Sx. (aggrevation after postop.)***	Sensory symptom at preoperation	-
**Preop. visual Sx. (aggrevation after postop.)***	Visual symptom at preoperation	-
**Preop. cognitive Sx. (aggrevation after postop.)***	Cognitive symptom at preoperation	-
**Motor (change to postop.)***	Preoperative motor symptom score	-
**KPS (change to postop.)***	Preoperative Karnofsky performance status	-
**OS time***	Overall survival time	days
**OS***	Overall survival	0; Alive, 1; Death
**PFS time***	Progression free survival time	days
**PFS***	Progression free survival	0; Stable, 1; Progression

Postop.; Post-operation
