# Supplementary material for: Maximal surgical resection and adjuvant surgical technique to prolong the survival of adult patients with thalamic glioblastoma
Source: PLoS One. 2021 Feb 4;16(2):e0244325. doi: 10.1371/journal.pone.0244325 (PMC7861362; doi:10.1371/journal.pone.0244325)
Supplement: S1 Fig — (DOCX) [file pone.0244325.s002.docx]

**S1 Fig.** KM-plots showing difference between surgical resection and each biopsy condition group with overall survival within the MGMT un-methylated group (a), and the methylated group (b)
