# Supplementary material for: Maximal surgical resection and adjuvant surgical technique to prolong the survival of adult patients with thalamic glioblastoma
Source: PLoS One. 2021 Feb 4;16(2):e0244325. doi: 10.1371/journal.pone.0244325 (PMC7861362; doi:10.1371/journal.pone.0244325)
Supplement: S2 Fig — (DOCX) [file pone.0244325.s003.docx]

**S2 Fig.** KM-plots showing difference between surgical resection and patient selecting biopsy group with overall survival and progression-free survival within the MGMT un-methylated group (a, b), and the methylated group (c, d)
