# Supplementary material for: Maximal surgical resection and adjuvant surgical technique to prolong the survival of adult patients with thalamic glioblastoma
Source: PLoS One. 2021 Feb 4;16(2):e0244325. doi: 10.1371/journal.pone.0244325 (PMC7861362; doi:10.1371/journal.pone.0244325)
Supplement: S4 Fig — (DOCX) [file pone.0244325.s005.docx]

**S4 Fig.** KM-plots showing difference in overall survival (a) and progression-free survival (b) between GTR and STR within the surgical resection only group, overall survival (c) and progression-free survival (d) between the STR group and the biopsy group.
