# Supplementary material for: Maximal surgical resection and adjuvant surgical technique to prolong the survival of adult patients with thalamic glioblastoma
Source: PLoS One. 2021 Feb 4;16(2):e0244325. doi: 10.1371/journal.pone.0244325 (PMC7861362; doi:10.1371/journal.pone.0244325)
Supplement: S5 Fig — (DOCX) [file pone.0244325.s006.docx]

**S5 Fig.** KM plots of comparison in terms of overall (a) or progression-free (b) survival between patients who were above and below at 30 years old within the surgical resection group
