# Supplementary material for: Maximal surgical resection and adjuvant surgical technique to prolong the survival of adult patients with thalamic glioblastoma
Source: PLoS One. 2021 Feb 4;16(2):e0244325. doi: 10.1371/journal.pone.0244325 (PMC7861362; doi:10.1371/journal.pone.0244325)
Supplement: S6 Fig — Yes to No; score less than Grade 5 of motor symptom or presence of other symptoms at the preoperative state but improved after operation, Yes to Yes; score less than Grade 5 of motor symptom or presence of other symptoms at the preoperative state and no change in the state after operation, No to Yes; Grade 5 of motor symptom or without other symptoms at the preoperative state but occurred after operation, No to No; Grade 5 of motor symptom or without any another symptom at both the pre- and postoperative states. (DOCX) [file pone.0244325.s007.docx]

**S6 Fig.** Pie charts of motor (a), cognitive (b), visual (c) or sensory symptom (d) change from the preoperative status to the postoperative status. Yes to No; score less than Grade 5 of motor symptom or presence of other symptoms at the preoperative state but improved after operation, Yes to Yes; score less than Grade 5 of motor symptom or presence of other symptoms at the preoperative state and no change in the state after operation, No to Yes; Grade 5 of motor symptom or without other symptoms at the preoperative state but occurred after operation, No to No; Grade 5 of motor symptom or without any another symptom at both the pre- and postoperative states
