# Supplementary material for: Maximal surgical resection and adjuvant surgical technique to prolong the survival of adult patients with thalamic glioblastoma
Source: PLoS One. 2021 Feb 4;16(2):e0244325. doi: 10.1371/journal.pone.0244325 (PMC7861362; doi:10.1371/journal.pone.0244325)
Supplement: S7 Fig — (DOCX) [file pone.0244325.s008.docx]

**S7 Fig.** KM plots showing overall survival curves of postoperative sensory (a), motor (b), visual (c), and cognitive (d) symptom worsening
