# Supplementary material for: Maximal surgical resection and adjuvant surgical technique to prolong the survival of adult patients with thalamic glioblastoma
Source: PLoS One. 2021 Feb 4;16(2):e0244325. doi: 10.1371/journal.pone.0244325 (PMC7861362; doi:10.1371/journal.pone.0244325)
Supplement: S9 Fig — Preoperative and postoperative magnetic resonance image of a glioblastoma of the right posterior thalamus with lateral extension (A, B). Fluorescence dye (5-aminolevulinic acid) imaging of the posterior thalamus with lateral extension (C, D). Preoperative magnetic resonance image was fused with an intraoperative computed tomography image and use of the tailed bullet technique during the operation for enabling adjustment for brain shifting and the confirmation of target lesion (E, F). (DOCX) [file pone.0244325.s010.docx]

**S9 Fig.** Preoperative and postoperative magnetic resonance image of a glioblastoma of the right posterior thalamus with lateral extension (A, B). Fluorescence dye (5-aminolevulinic acid) imaging of the posterior thalamus with lateral extension (C, D). Preoperative magnetic resonance image was fused with an intraoperative computed tomography image and use of the tailed bullet technique during the operation for enabling adjustment for brain shifting and the confirmation of target lesion (E, F)
